# Small Bowel Ischemia and Ischemic Colitis Secondary to Thrombotic Thrombocytopenic Purpura in a Patient With Systemic Lupus Erythematous: A Rare Clinical Entity

**DOI:** 10.7759/cureus.65666

**Published:** 2024-07-29

**Authors:** Hazem Abosheaishaa, Vincent Rizzo, Muhammad Haseeb ul Rasool, Mahmoud Nassar, Khaled A Elfert, Saphwat Eskaros

**Affiliations:** 1 Internal Medicine, Icahn School of Medicine at Mount Sinai, Queens Hospital Center, New York, USA; 2 Internal Medicine/Gastroenterology, Cairo University, Cairo, EGY; 3 Medicine, Icahn School of Medicine at Mount Sinai, Queens Hospital Center, New York, USA; 4 Endocrinology, Diabetes, and Metabolism, Jacobs School of Medicine and Biomedical Sciences, University at Buffalo, Buffalo, USA; 5 Internal Medicine, St. Barnabas Hospital Health System, New York, USA; 6 Gastroenterology, Icahn School of Medicine at Mount Sinai, Queens Hospital Center, New York, USA

**Keywords:** thrombotic thrombocytopenic purpura (ttp), systemic lupus erythematosus (sle), gi bleeding, ischemic colitis, small bowel ischemia

## Abstract

Thrombotic thrombocytopenic purpura (TTP) is a rare, life-threatening hematologic disorder characterized by microangiopathic hemolytic anemia, thrombocytopenia, and organ dysfunction. This report highlights a rare case of small bowel ischemia and ischemic colitis caused by TTP in a 35-year-old woman with systemic lupus erythematosus (SLE), hypertension, and end-stage renal disease on hemodialysis. She presented with severe abdominal pain, diarrhea, vomiting, and bloody bowel movements. Diagnosed through CT, EGD, and colonoscopy and confirmed by ADAMTS13 levels, she was treated with plasma exchange, steroids, and rituximab. After standard therapies failed, resection anastomosis surgery led to clinical improvement. This case underscores the importance of early recognition and treatment of TTP in SLE patients to improve prognosis and reduce morbidity and mortality.

## Introduction

Thrombotic thrombocytopenic purpura (TTP) is a rare and potentially fatal hematological disorder that is characterized by microangiopathic hemolytic anemia, thrombocytopenia, and organ dysfunction due to widespread microvascular platelet-rich thrombi [1]. TTP can either be idiopathic or secondary to various clinical conditions, such as autoimmune disorders, infections, transplants, neoplasia, drugs, and pregnancy [2]. Although there is a significant overlap in the presenting features of TTP and systemic lupus erythematosus (SLE), TTP is not recognized as a criterion for the classification of SLE, and only a minority of SLE patients have concurrent TTP [3]. Autopsy studies have suggested that TTP may be underdiagnosed in SLE due to similarities in clinical manifestations [4]. This case report underscores a rare presentation of small bowel ischemia and ischemic colitis secondary to TTP in a patient with a known history of SLE, thereby emphasizing the importance of considering TTP as a potential cause of acute abdominal symptoms in SLE patients.

## Case presentation

A 35-year-old female patient with hypertension, SLE, end-stage renal disease (ESRD), and regular hemodialysis (HD) presented to the emergency department (ED) with abdominal pain, which had started the previous night and had worsened since then. She described the pain as diffuse, generalized, severe, and unrelated to meals, with no identifiable precipitating or alleviating factors. Despite experiencing pain, she reported a decreased appetite but denied having nausea, vomiting, changes in bowel movements, dark stools, and rectal bleeding. She also denied any recent travel or illness, and a review of her systems was unremarkable. On examination, she was afebrile, hypertensive, and tachycardic and had normal oxygen saturation. Physical examination revealed diffuse abdominal tenderness.

The patient had been diagnosed with SLE at the age of 25 based on positive ANA, low complement, alopecia, enteritis, and recurrent pericarditis. Over the past 12 years, she had required multiple tapers of prednisone and was being treated with hydroxychloroquine. She had a significant obstetric history, including seven miscarriages, with one live birth; however, the baby died shortly after birth. She had been on aspirin and apixaban, with aspirin being discontinued a year prior to this presentation.

**Figure 1:**
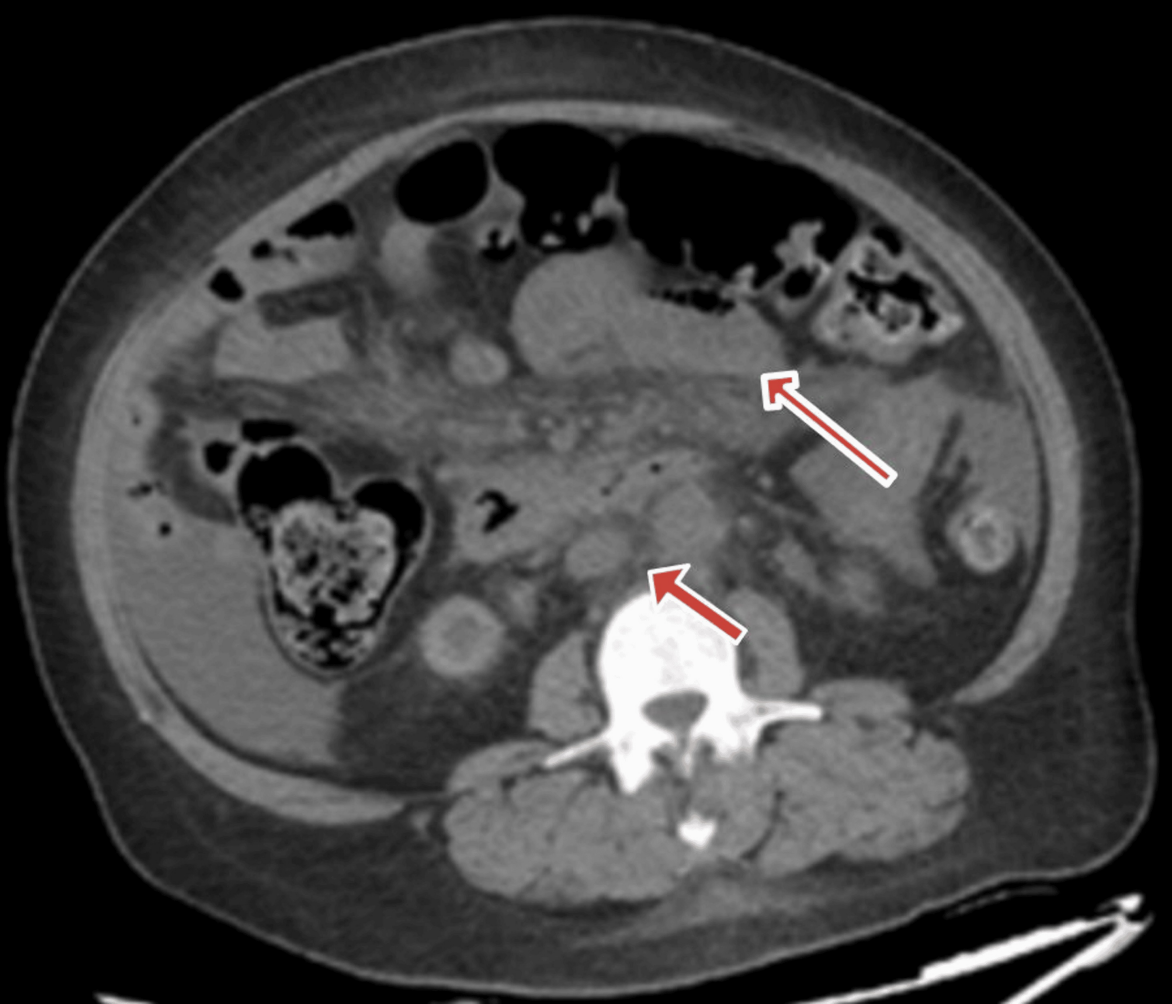
CT scan showing mesenteric lymphadenopathy and close-loop obstruction

The patient was treated in the ED with normal saline, morphine, and famotidine. The results of the blood work are listed below (Table 1). CT of the abdomen and pelvis without contrast revealed moderate ascites, celiac, and mesenteric adenopathy and concern for a closed-loop obstruction (Fig. 1). General surgery was consulted, and a CT scan of the abdomen with oral and intravenous contrast was recommended. No acute findings were found on the CT scan. The patient was admitted for further workup.

**Table 1:** Laboratory results at the time of admission BUN: blood urea nitrogen, DRVVT: dilute Russell viper venom time, ALT (SGPT): alanine transferase (serum glutamic-pyruvic transaminase), AST (SGOT): aspartate transaminase (serum glutamic oxaloacetic transaminase), eGFR: estimated glomerular filtration rate, HCG: human chorionic gonadotropin, PTH: parathyroid hormone, LDH: lactate dehydrogenase

Lab (normal value)	Result
Hemoglobin (12.0-16.0 g/dL)	6.6
White blood cell (4.8010.80 x 10(3)/mcl)	7.72
Red blood cell (4.20-5.4080 x 10(6)/mcl)	2.34
Platelets (150-450 x 10(3)/mcl)	52
Sodium (136-145 mmol/L)	137
Potassium (3.5-5.1 mmol/L)	4.4
Chloride (98-108 mmol/L)	92
CO_2_ (22-29 mmol/L)	27
BUN (6-23 mg/dL)	65
Creatinine (0.50-1.20 mg/dL)	10.94
Calcium (8.6-10.3 mg/dL)	77
Albumin (3.5-5.2 g/dL)	3.1
Total protein (6.6-8.7 g/dL)	8.1
Total bilirubin (0.0-1.2 mg/dL)	0.5
Alkaline phosphatase (35-104 U/L)	127
DRVVT 50/50 (<1.2 seconds)	42.0
Cardiolipin antibodies (negative)	Negative
Haptoglobin (34-200 mg/dL)	99
ALT (SGPT) (0-33 U/L)	9
AST (SGOT) (5-32 U/L)	21
Anion gap (8-16 mEq/L)	18
eGFR (cr) (>60 ml/min/1.73 m^2^)	4
pH venous (7.32-7.43)	7.57
pCO_2_ venous (38-41 mmHg)	32
pO_2_ venous (30-50 mmHg)	54
Lactate venous (0.6-1.4 mmol/L)	1.1
Troponin T (<0.010 ng/mL)	0.062
Lipase (13-60 U/L)	44
HCG quantitative (<5.0mIU/mL)	<1.0
Phosphorus (2.5-4.5 mg/dL)	6.2
PTH intact (15-65 pg/mL)	1805
Magnesium (1.60-2.60 mg/dL)	2.10
Ferritin (15-150 ng/mL)	3449
LDH (135-214 U/L)	625
Fibrinogen (200-393 mg/dL)	380
D-dimer (<230 ng/ml)	6563

**Figure 2:**
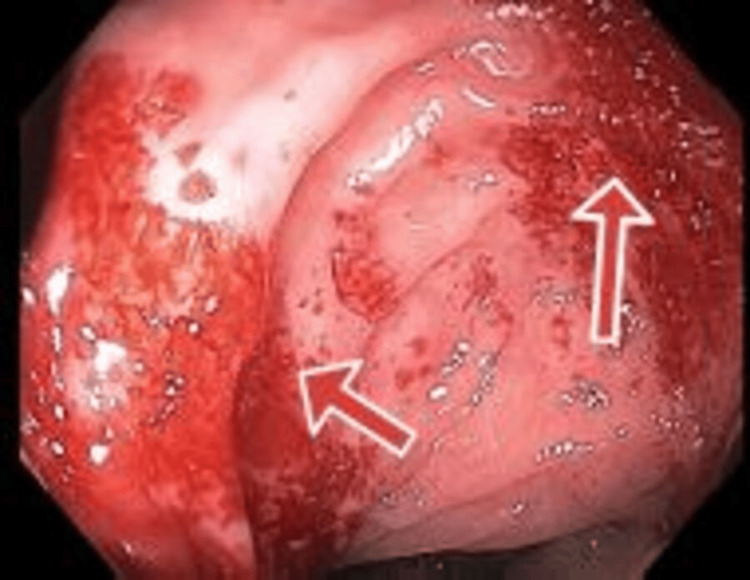
Ascending colon and hepatic flexure showing non-bleeding ulcers

Post-admission, the patient was evaluated for microcytic hypochromic anemia and received two packed RBCs; however, she reported having two red bloody loose bowel movements. The hematologist was consulted for the present anemia and thrombocytopenia and recommended dexamethasone daily and blood work for possible immune thrombocytopenic purpura (ITP) in the setting of SLE. For possible lupus flare-ups, rheumatology was consulted, and blood work was recommended; in addition, gastroenterology was consulted for abdominal pain and bloody diarrhea. Three days later, esophagogastroduodenoscopy (EGD) and colonoscopy were performed as per gastrointestinal recommendations. They revealed non-bleeding ulcers at the duodenum and gastric antrum and a discontinuous area of non-bleeding ulcerated mucosa with exudate at the ascending colon and hepatic flexure, respectively (biopsies were taken for histopathology) (Fig. 2).

**Figure 3:**
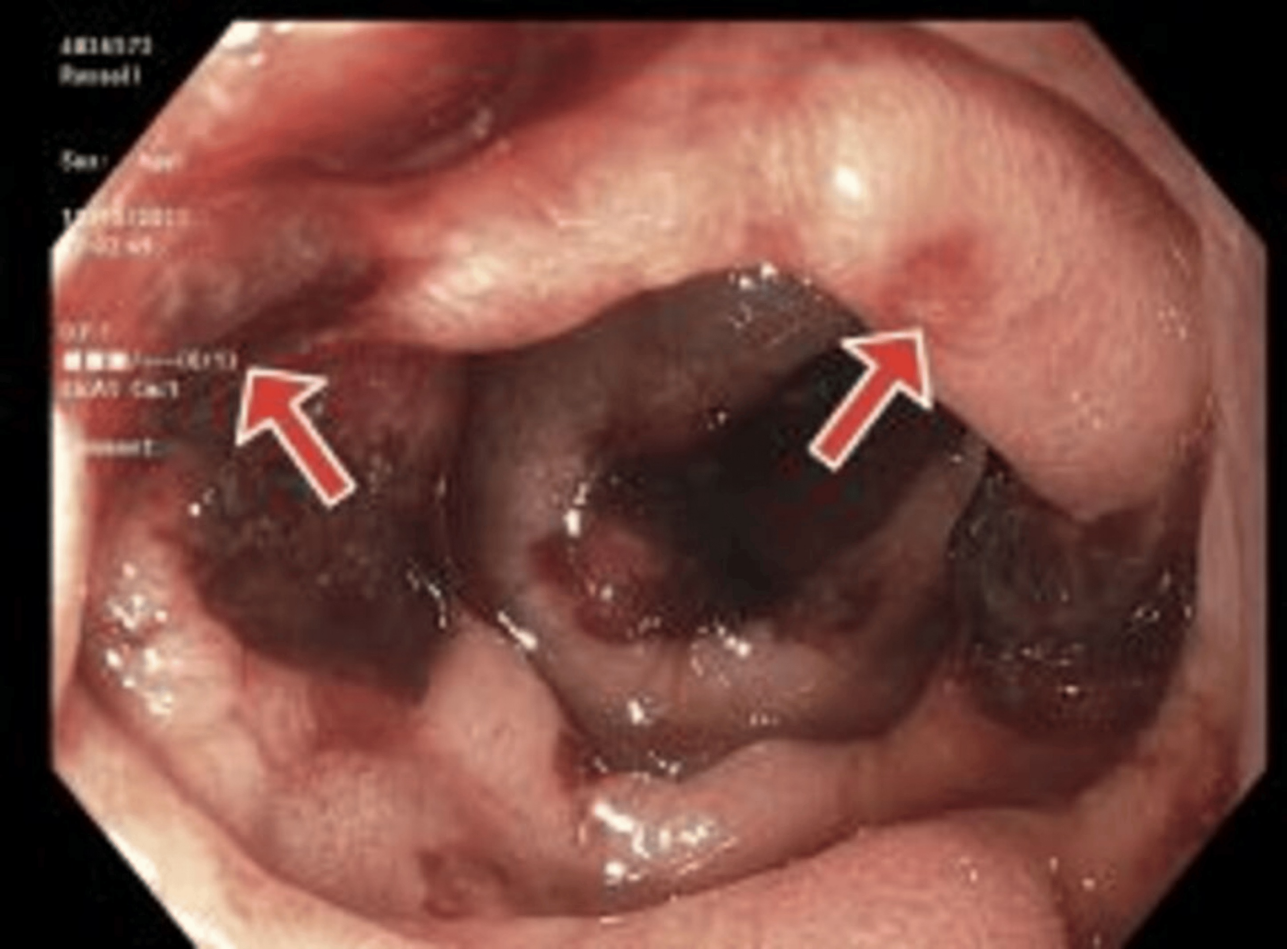
Pre-pyloric gastric mucosa showing gastric ulcers with dusky mucosa and non-bleeding ulcers

A follow-up EGD and small intestine enteroscopy three days later revealed erosive gastritis with duodenal and jejunal ulcerations (Fig. 3). As a result of diffuse gastrointestinal (GI) ulceration and thrombocytopenia, the patient's hemoglobin dropped to 5.2, and she was transferred to the Intermediary care unit for blood transfusion and stabilization. A hematologist recommended switching dexamethasone to oral high-dose prednisolone and initiating intravenous immunoglobulin (IVIG) for suspected immune-mediated thrombocytopenia. Follow-up labs are shown below (Table 2).

**Table 2:** Follow-up laboratory results BUN: blood urea nitrogen, APTT: activated partial thromboplastin time, ENA: extractable nuclear antigen, AST (SGOT): aspartate transaminase (serum glutamic oxaloacetic transaminase), eGFR: estimated glomerular filtration rate, LDH: lactate dehydrogenase, RNP: ribonucleoprotein

Lab (normal value)	Result
Hemoglobin (12.0-16.0 g/dL)	5.6
White blood cell (4.8010.80 x 10(3)/mcl)	42.46
Red blood cell (4.20-5.4080 x 10(6)/mcl)	1.99
Platelets (150-450 x 10(3)/mcl)	14
Sodium (136-145 mmol/L)	135
Potassium (3.5-5.1 mmol/L)	5.7
Chloride (98-108 mmol/L)	96
CO_2_ (22-29 mmol/L)	22
BUN (6-23 mg/dL)	106
Creatinine (0.50-1.20 mg/dL)	5.79
Calcium (8.6-10.3 mg/dL)	8.9
Albumin (3.5-5.2 g/dL)	2.5
Total protein (6.6-8.7 g/dL)	5.4
APTT 100% (27.5-35.5 sec)	25.3
ENA Smith AB (<0.9 AI)	<0.2
C3 (81-157 mg/dL)	113
ADAM T13 activity (>66.8%)	3.0
Total bilirubin (0.0-1.2 mg/dL)	0.7
Alkaline phosphatase (35-104 U/L)	92
ALT (SGPT) (0-33 U/L)	9
AST (SGOT) (5-32 U/L)	23
Anion gap (8-16 mEq/L)	17
eGFR (cr) (>60 ml/min/1.73 m^2^)	9
LDH (135-214 U/L)	701
Fibrinogen (200-393 mg/dL)	386
Haptoglobin (34-200 mg/dL)	145
Reticulocytes (0.50-1.50%)	3.36
Direct antiglobulin test (Negative)	Negative
Calprotectin (stool) (0-120 ug/g)	1507
Fibrinogen (200-393 mg/dL)	372
Anti-dsDNA Ab (<29 IU/mL)	19
ENA RNP Ab (<0.9 AI)	4.5
C4 (13-39 mg/dL)	30
Haptoglobin (34-200 mg/dL)	181

Based on the low ADAMTS13 and the presence of schistocytes in the peripheral smear, the patient was diagnosed with TTP. Plasmapheresis was recommended after the patient had received five doses of IVIG without significant improvement in the platelet count. Despite slow improvements, plasmapheresis continued for 14 sessions until the platelet count was normal. After the 14 sessions of plasma exchange, the ADAMTS13 activity was 68.3%, so plasma exchange was stopped.

**Figure 4:**
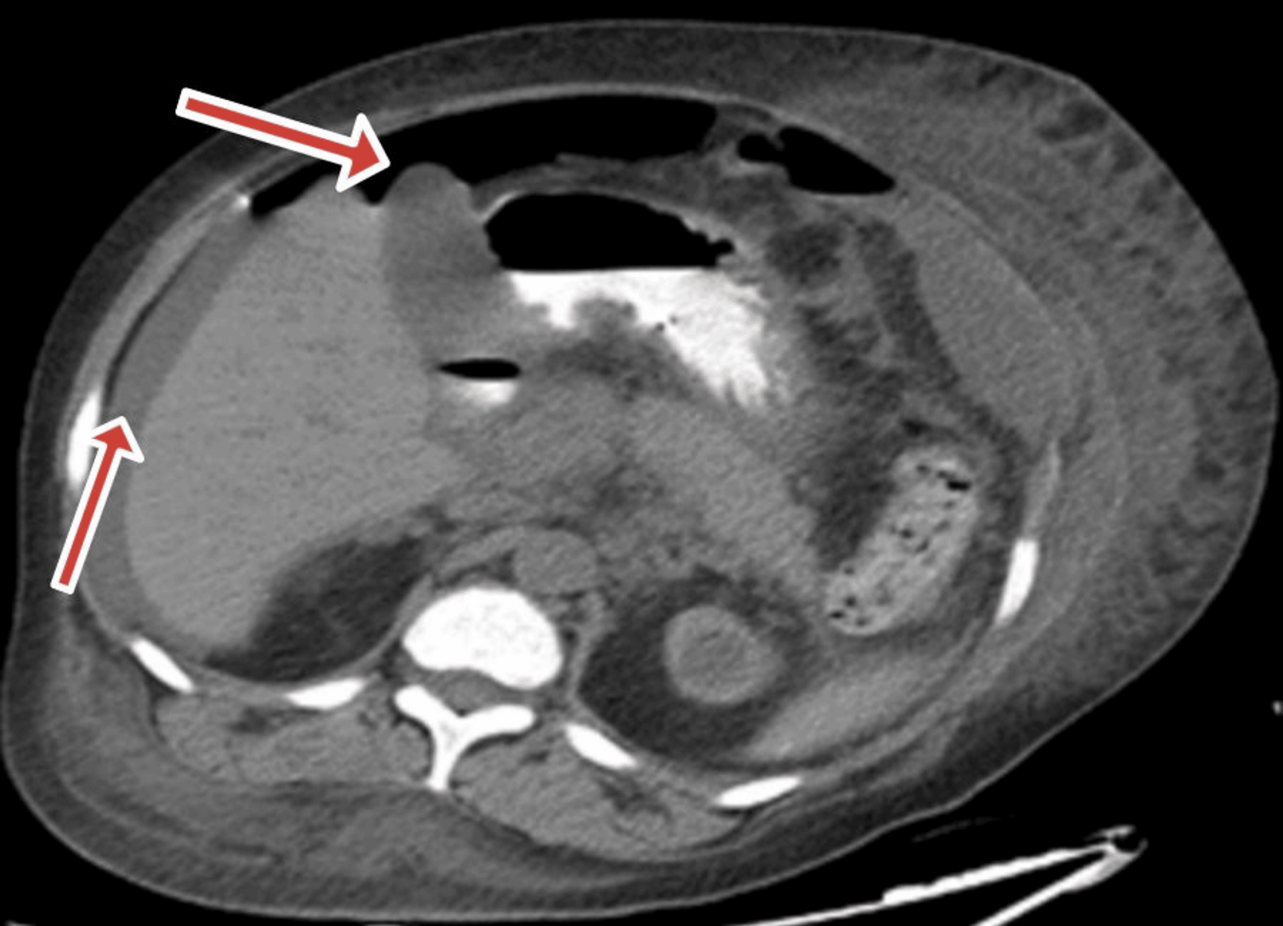
CT abdomen and pelvis showing a small-volume pneumoperitoneum, moderate ascites, mild pleural effusion, and retroperitoneal lymph nodes

**Figure 5:**
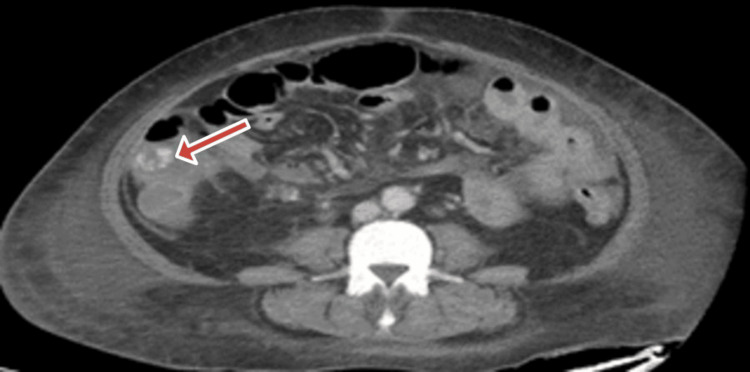
CT abdomen and pelvis, showing active contrast extravasation in ascending colon (the white spot on the right upper corner)

However, despite normal platelet counts, the patient experienced severe abdominal pain and rectal bleeding. A CT scan of the abdomen and pelvis revealed a small volume pneumoperitoneum, moderate ascites, mild pleural effusion, and retroperitoneal lymph nodes (Fig. 4). An ultrasound-guided ascitic fluid sample revealed *Klebsiella* and *Candida*, likely secondary to intraperitoneal infections caused by bowel perforation, for which meropenem was prescribed. Resulting from repeated episodes of gastrointestinal bleeding, tagged RBC scintigraphy was conducted and revealed acute bleeding in the ascending colon. Computed tomography angiography (CTA) (Fig. 5) revealed persistent pneumoperitoneum. Lab tests revealed elevated white counts and elevated liver enzymes (alanine transaminase (ALT) = 265, aspartate transaminase (AST) = 423), indicating that the liver had been shocked as a result of low blood pressure and sepsis.

After 48 hours, the patient was weaned off the ventilator, reported relief from abdominal pain, passed flatus, and tolerated fluid intake. The results of the laboratory tests revealed an improvement in the white blood cell (WBC) count. Despite the correction of electrolytes, there was minimal output in the ileostomy; however, the mucosa was healthy on examination. The patient continued to have melena through the ileostomy, causing a drop in hemoglobin and requiring serial transfusion. Interval analysis of the peripheral smear revealed the reappearance of schistocytes, for which she was restarted on plasma exchanges. CTA was performed again to locate the source of bleeding, which demonstrated intramural bleeding around the site of anastomosis. The patient was hemodynamically stable, had multiple comorbidities, and was at risk of preoperative bleeding, so a multidisciplinary team consisting of a hematologist, surgeon, and critical care team decided against surgery and preferred to involve Interventional radiology. The patient underwent angiography followed by embolization of the bleeding branch of the superior mesenteric artery. However, the procedure was complicated by the formation of a pseudoaneurysm and localized bleeding at the site of catheter insertion. The patient continued to require daily blood products for possible disseminated intravascular coagulation (DIC) in the setting of TTP and anemia. An interval blood workup was performed for the alternative reasons of the presentation, and the results are shown below (Table 3).

**Table 3:** Differential diagnosis work-up CEA: carcinoembryonic antigen, AFP: alpha-fetoprotein, IGM: immunoglobulin M, CMV PCR: cytomegalovirus polymerase chain reaction, DS DNA AB: double stranded DNA antibody

Lab (normal value)	Results
CEA (0.0-3.8 ng/mL)	2.2
AFP (<8.3 ng/mL)	<1.8
ADAMTS activity (>66.8%)	22.4
Heparin PF4 AB (0.0-0.9 U/mL)	<0.6
Babesia IgG (<1:10)	Neg <1:10
*Anaplasma phagocytophilum*, *Ehrlichia chaffeensis*, *Ehrlichia ewingii* (negative)	Negative
CA125 (<38 U/mL)	32
CA 19-9 (<35 U/mL)	32
Serotonin release assay (less than 20%)	0%
Babesia IGM (<1:10)	<1:10
CMV PCR (<0IU/mL)	3360
DS DNA AB (negative)	Negative

Vascular surgery assessed the patient and performed ultrasound-guided decompression of the pseudoaneurysm. The patient continued to require serial blood products for low hemoglobin and fibrinogen while receiving daily plasma exchange. After a multidisciplinary discussion, it was planned for the GI to perform a push enteroscopy to localize the site of ongoing bleeding. Enteroscopy demonstrated active bleeding around the first jejunal anastomosis, for which the initial application of a hemostasis clip failed. Epinephrine was injected, followed by cauterization to achieve hemostasis. There was a cratered ulcer in the duodenum, with no evidence of acute bleeding. After the procedure, the patient's hemoglobin stabilized. The hemorrhagic output from the ileostomy settled, and she did not require a blood transfusion after that. The interval ADAM13 essay was within normal limits, and there were no schistocytes on the peripheral smear, so the plasma exchange was stopped. Total parenteral nutrition (TPN) was held, and the patient gradually escalated on oral intake while kept on peripheral parenteral nutrition (PPN). With the improving TTP, the hematologist decided to start her on rituximab to keep the TTP in remission. Prior to starting the immune suppression, an initial workup was performed to confirm the absence of infectious disease. The patient was seen one week and one month after at the hematology clinic in stable condition.

## Discussion

Moschcowitz was the first to identify a case of TTP in a 16-year-old female who acutely complained of high fever, weakness, pain, pallor, and petechiae in 1924. A few days later, she had neurological conditions, became comatose, and then passed away; her microcirculation autopsy showed disseminated hyaline in the liver, kidney, spleen, and heart [5]. In 1985, histopathological studies concluded that visceral microthrombi secondary to TTP is primarily composed of platelets and von Willebrand factor (VWF) thrombi, rather than fibrinogen [6]. A deficiency in a protease specifically able to cleave VWF, called ADAMTS13, was linked to the pathophysiology of TTP [7]. The exclusive biological marker for TTP is ADAMTS13, and TTP diagnosis requires testing for ADAMTS13 [1]; due to a persistent or recurrent ADAMTS13 deficiency, TTP patients have a tendency to relapse [8]. 

TTP can be idiopathic or secondary to a number of clinical conditions, including autoimmune disorders, infections, hematopoietic stem cell or solid organ transplants, neoplasia, drugs, and pregnancy, and it can be difficult to differentiate between other thrombotic microangiopathy (TMA) syndromes, cancers, or autoimmune diseases [2]. Acquired TTP development has been linked to genetic factors. Obesity, black ethnicity (African Americans or Africans from the Caribbean), and female gender have all been shown to be associated with the development of anti-ADAMTS13 autoantibodies [1].

In 2016, Takimoto et al. [9] reported a case of TTP initially presented with acute myocardial infarction (AMI). The pancreas was found to be one of the primary organs revealing microscopic microthrombi, according to a large autopsy series involving 25 patients with TMA secondary to TTP [10]. See et al. reported a case of acute ischemic colitis as a typical presentation of TTP [11]. Interestingly, Abeysundara et al. described a case of jejunal stricture as a rare presentation of TTP in 2017 [12]. 

TTP has been associated with macrovascular thrombosis; for instance, in a group of 55 patients who were admitted to the ICU for TMA secondary to TTP, half (n = 28) had venous or arterial vascular thrombosis, including seven (12.7%) patients who had cerebral artery thrombosis and 21 (38%) patients who had deep vein thrombosis (13 (23.6%) of them had central venous catheters) [13]. 

There is a significant overlap between the presenting features of TTP and SLE, although TTP is not recognized as a criterion for the classification of SLE, and a small number of SLE patients also have concurrent TTP [3]. TMA has been thought to only occasionally occur in people with SLE. Autopsy studies have suggested that TMA may be underdiagnosed in SLE because of the similarity in symptoms, but there has been an increase in the reporting of this link in recent years. Nesher et al. [4] reported four cases with SLE-associated TMA in their case series and described another 24 cases from the literature. 

In 2016, Abu-Hishmeh et al. [14] reported a case of resistant TTP and SLE, who is a 34-year-old female who failed initial therapy with plasmapheresis, high-dose steroids, and rituximab but showed improvement with cyclophosphamide.

The standard therapy for TTP remains plasma exchange, steroids, and rituximab. Recently, another monoclonal antibody caplacizumab that targets the A1 domain of the ADAMTS13 inhibiting interaction between platelets and VWF multimers and a recombinant ADAMTS13 are being investigated in clinical trials as a potential therapy for TTP [8].

Up to our knowledge, this is the first case in the literature to report small bowel ischemia with ischemic colitis secondary to TTP in the setting of the known history of SLE. Hence, we aim to raise awareness about this clinical scenario as a common presentation of TTP.

## Conclusions

This case report presents a rare clinical presentation of small bowel ischemia and ischemic colitis secondary to TTP in a patient with a known history of SLE. This highlights the importance of maintaining a high index of suspicion for TTP in SLE patients presenting with acute abdominal symptoms. Early recognition and prompt initiation of appropriate therapy, including plasma exchange, steroids, and immunosuppressive agents, can significantly improve the prognosis and reduce the morbidity and mortality associated with TTP. Clinicians should be aware of the potential overlap between TTP and SLE and the broad range of clinical manifestations that can occur in these patients. This case emphasizes the need for increased awareness and prompt diagnosis to optimize patient outcomes and reduce the risk of severe complications.
